# Protective efficacy and safety of liver stage attenuated malaria parasites

**DOI:** 10.1038/srep26824

**Published:** 2016-05-31

**Authors:** Hirdesh Kumar, Julia Magdalena Sattler, Mirko Singer, Kirsten Heiss, Miriam Reinig, Christiane Hammerschmidt-Kamper, Volker Heussler, Ann-Kristin Mueller, Friedrich Frischknecht

**Affiliations:** 1 Integrative Parasitology Center for Infectious Diseases, Heidelberg University Medical School, Im Neuenheimer Feld 324, 69120 Heidelberg, Germany; 2 Parasitology, Center for Infectious Diseases, Heidelberg University Medical School, Im Neuenheimer Feld 324, 69120 Heidelberg, Germany; 3 MalVa GmbH Heidelberg, Germany; 4 Institute of Cell Biology, Balzerstrasse 4, 3012 Bern, Switzerland; 5 German Center for Infectious Diseases, Im Neuenheimer Feld 324, 69120 Heidelberg, Germany

## Abstract

During the clinically silent liver stage of a *Plasmodium* infection the parasite replicates from a single sporozoite into thousands of merozoites. Infection of humans and rodents with large numbers of sporozoites that arrest their development within the liver can cause sterile protection from subsequent infections. Disruption of genes essential for liver stage development of rodent malaria parasites has yielded a number of attenuated parasite strains. A key question to this end is how increased attenuation relates to vaccine efficacy. Here, we generated rodent malaria parasite lines that arrest during liver stage development and probed the impact of multiple gene deletions on attenuation and protective efficacy. In contrast to *P. berghei* strain ANKA *LISP2*(–) or *uis3*(–) single knockout parasites, which occasionally caused breakthrough infections, the double mutant lacking both genes was completely attenuated even when high numbers of sporozoites were administered. However, different vaccination protocols showed that *LISP2*(–) parasites protected better than *uis3*(–) and double mutants. Hence, deletion of several genes can yield increased safety but might come at the cost of protective efficacy.

Malaria is transmitted through the bite of a mosquito, which injects *Plasmodium* sporozoites into the host. After migration through the skin, sporozoites enter the blood stream and invade hepatocytes to further differentiate into merozoites, which in turn infect red blood cells causing the clinical symptoms of malaria. Successful experimental vaccines that protect from natural infection have first been generated by radiation treatment of *Plasmodium* sporozoites^1^. These damaged parasites could still enter hepatocytes but, at the right doses of radiation, did not replicate and failed to develop into merozoites^2^. When applied in large numbers these defective sporozoites could induce protective immunity from subsequent wild type sporozoite challenge in both rodents and humans^1^,^3^,^4^. Similarly, infection of humans and rodents with wild type sporozoites followed by application of parasite-targeting drugs also generated protective immunity^5^,^6^,^7^,^8^. Ablation of genes that are essential for liver stage development has expanded the potential for generating experimental whole parasite vaccines, generally called genetically attenuated parasites, GAPs^9^,^10^,^11^,^12^,^13^,^14^. GAPs arresting late during development were found to confer a stronger protective immunity in rodent models potentially due to a broader antigenic repertoire and/or prolonged persistence^15^. However, independent from the time of arrest GAPs can still cause breakthrough infections such that rodents or humans develop a blood stage infection^16^,^17^,^18^. Thus, in order to produce safer GAPs, multiple genes have been deleted from the respective *Plasmodium* genomes^19^,^20^,^21^,^22^. Yet, all of the combined gene deletions lead to an arrest early during liver stage development. While several rodent GAPs have been developed that confer long lasting immunity, this has not yet been completely translated into a genetically attenuated human malaria parasite^21^. One important difference between the species being that human malaria parasites take over a week to fully develop in hepatocytes, while rodent parasites do so in less than three days^23^. In a first trial with *P. falciparum* GAPs, ablation of at least three genes appeared to be necessary to achieve complete arrest^21^. Yet, how ablation of multiple genes affects vaccine efficacy is not clear. Thus more candidate genes need to be identified and tested in rodent malaria parasites that could then be translated to generate efficient attenuated *P. falciparum* strains^24^.

Here we investigated *Plasmodium berghei* parasites lacking a gene, *LISP2* (for liver specific protein 2), which is specifically expressed in liver stages^25^ and exported from the parasite to the host cell^26^. The partial disruption of this gene yielded severely impaired, but not completely attenuated liver stages^26^,^27^. For this reason the gene would not be listed as a potential GAP candidate. However, we now show that complete disruption of *LISP2* caused a growth arrest during liver stage development and yielded a genetically attenuated rodent malaria parasite line. Additional depletion of the *uis3* (for up-regulated in infectious sporozoites 3) gene in *P. berghei* strain ANKA increased the safety of the vaccination strain. However, the level of protection induced by this double knockout strain was lower than that obtained with single *LISP2*(–) knockout strain. Interestingly, we also observed a marked parasite strain-specific difference upon depletion of the *uis3* gene between *P. berghei* strains NK65 and ANKA. In *P. berghei* NK65, deletion of *uis3* led to early and complete arrest during liver stage development^9^. Curiously *uis3*(–) ANKA parasites showed a different phenotype as they were not completely attenuated and arrested later in development than *uis3*(–) NK65 and *P. yoelii* parasites, respectively^28^. This allowed us to test two attenuated parasites and a parasite lacking both genes for attenuation and vaccination efficacy.

## Results

### *P. berghei* strain ANKA sporozoites lacking *uis3* arrest during mid-liver stage development and can cause breakthrough infections

**Figure 1:**
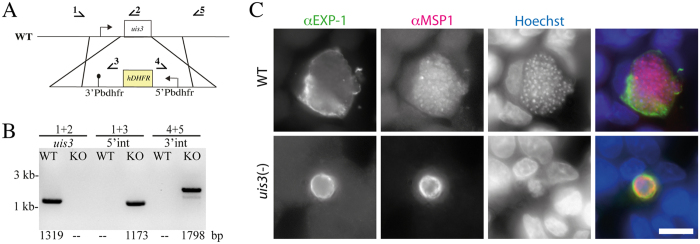
Generation and characterization of *P. berghei* ANKA *uis3*(–) parasites. (**A**) Schematic of the *uis3* replacement strategy using the 5′ and 3′UTRs of *uis3* to integrate a resistance cassette by double crossover into the *P. berghei* strain ANKA genome. Locations of primers used for PCR in (**B**) are indicated. (**B**) Diagnostic PCR to investigate generation of *uis3*(–) parasites. WT: wild type, KO: *uis3*(–) parasite line. Numbers below the gel indicate expected amplicon sizes. (**C**) Liver stages in HepG2 cells formed by WT and *uis3*(–) sporozoites 65 hours post infection labeled with antibodies against EXP-1 and MSP1; Hoechst reveals host and parasite DNA. Images on the right show the merged images. Scale bar: 10 μm.

*P. berghei* parasites of the NK65 strain that lack *uis3* arrest early during hepatic development, yet they confer complete protection from subsequent infection in a classical 3-shot vaccination regimen^9^. The *P. berghei* ANKA strain undergoes a more rapid development in the liver stage than *P. berghei* NK65^29^,^30^. To investigate potential differences in development and protective capacity of NK65 and ANKA, we generated an ANKA parasite line that lacks *uis3* (Fig. 1A,B). Like in the NK65 strain we could not detect any differences in blood stage or early mosquito stage development and sporozoites accumulated in similar numbers in mosquitoes as they did for the wild type (data not shown). Unexpectedly, the ANKA *uis3*(–) parasites developed into larger hepatic stages than shown for NK65 *uis3*(–) parasites^9^ as revealed by infection of HepG2 cells (Fig. 1C). Yet, their development was attenuated compared to wild type controls as the parasites did not form mature merozoites within 65 h as shown by altered merozoite surface protein 1 (MSP1) staining restricted to the surrounding parasite plasma membrane (Fig. 1C). However, ANKA *uis3*(–) sporozoites caused breakthrough infections in 6 out of 46 mice infected with a total of over 2.4 million sporozoites (Supplementary Table S2). Genotyping of these blood stage parasites confirmed that they were *uis3*(–) (data not shown). We also immunized mice intravenously with 50.000 ANKA *uis3*(–) sporozoites followed by two boosts with 20.000 ANKA *uis3*(–) sporozoites in a 3-dose regimen^9^. Mice with breakthrough infection were excluded from the experiment. When blood stage parasite free mice were challenged 14 days later with 10.000 wild type sporozoites all mice were protected (Table 1) as was reported for the NK65 *uis3*(–) parasites^9^.

### *P. berghei* strain ANKA sporozoites lacking *LISP2* arrest during liver stage development and cause rare breakthrough infection

**Figure 2:**
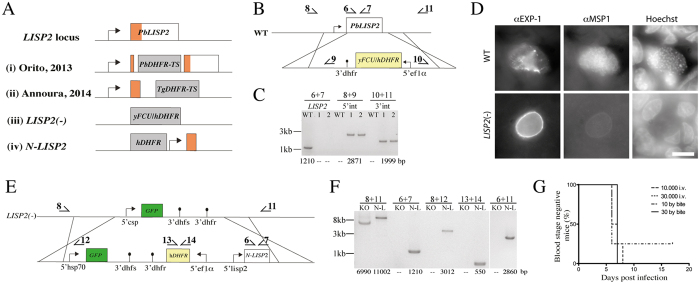
Generation and characterization of ANKA *LISP2*(–) and *N-LISP2* parasites. (**A**) *LISP2* locus and differently modified gene loci as generated in previous studies (i, ii) and in the current study (iii, iv). The region encoding the N-terminus is colored in orange. Boxes not drawn to scale. (**B**) Schematic of the *LISP2* replacement strategy using the 5′ and 3′UTRs of *LISP2* to integrate a resistance cassette by double crossover into the genome of the *P. berghei* strain ANKA. Note that this resistance cassette (yellow) included a negative selection marker to enable recycling of the cassette. The locations of primers used for PCR in (**C**) are indicated. (**C**) Diagnostic PCR to investigate generation of *LISP2*(–) parasites. Numbers below the gel indicate expected amplicon sizes. (**D**) Liver stages in HepG2 cells formed by ANKA WT and *LISP2*(–) sporozoites 65 hours post infection labeled with antibodies against EXP-1 and MSP1; Hoechst reveals host and parasite DNA. Scale bar: 10 μm. (**E**) Schematic of the strategy used to integrate the N-terminus of *LISP2* into the *LISP2(–)* parasite. The locations of primers used for PCR in (**F**) are indicated. The N-terminal fragment contained a start and stop codon. (**F**) Diagnostic PCR to investigate generation of *N-LISP2* parasites. Numbers below the gel indicate expected amplicon sizes. KO: *LISP2*(–) parasites, N-L: *N-LISP2* parasites. (**G**) Meyer-Kaplan plot to indicate the number of blood stage positive mice after injection of sporozoites i.v. and by bite. 4 groups of 4 mice each were infected; of these 15 became blood stage patent.

LISP2 is a 6-cys domain-containing protein with conserved gene structure across different *Plasmodium* species (Supplementary Figs S1 and S2). Previously, two laboratories generated *P. berghei* ANKA parasites lacking *LISP2*^26^,^27^. Curiously, both labs targeted the gene in a way that did not completely disrupt the gene (Fig. 2A). We thus generated a *P. berghei* ANKA parasite line lacking the entire coding sequence of LISP2 (Fig. 2B,C). Like in the previous reports we found no difference in blood stage development. ANKA *LISP2*(–) parasites infected mosquitoes at similar rates as wild type parasites and accumulated in the salivary glands at comparable numbers (data not shown). However, in contrast to the previously published results, we found that only one mouse out of 60 inoculated with a total of over 2.2 million ANKA *LISP2*(–) sporozoites developed a blood stage infection (Supplementary Table S2). Similar to ANKA parasites lacking *uis3*, we found that ANKA *LISP2*(–) parasites developed into large intrahepatic stages (Fig. 2D). Yet, immunofluorescence analysis of infected HepG2 cells revealed that there were no merozoites developing in ANKA *LISP2*(–) intrahepatic stages *in vitro*, similar to what was observed with ANKA *uis3*(–) parasites and little MSP1 was detectable in these parasites (Fig. 2D). After immunization of C57BL/6 mice with ANKA *LISP2*(–) sporozoites following the same 3-dose regimen as for ANKA *uis3*(–) sporozoites we also observed that all immunized mice were protected from subsequent challenge with wild type parasites (Table 1).

### The N-terminus of LISP2 is sufficient for full liver stage development

We next aimed to address the hypothesis that the N-terminus of LISP2 could account for the differences between our own (Fig. 2) and previous observations^26^,^27^. To this end we used negative selection^17^ to remove the resistance marker from one of the ANKA *LISP2*(–) parasite lines (Supplementary Fig. S3). Into the resulting parasite line we transfected a construct that led to the integration of the N-terminal 1210 base pairs of *LISP2*, which could still be expressed in one of the previously published parasite lines (Fig. 2E,F)^27^. We termed this parasite line ANKA *N-LISP2*. As expected and already observed with the previous *LISP2*(–) parasite lines the development of these parasites in the blood and mosquito stages was normal. When mice were infected with 10.000 *N-LISP2* sporozoites intravenously or by the bites of 10 mosquitoes we found that 15 out of 16 mice developed a blood stage infection, albeit with a delayed prepatency (Fig. 2G and Supplementary Table S2). These data show that the N-terminus of LISP2 can partially rescue the defect in liver stage development of ANKA *LISP2*(–) parasites.

### Parasites lacking *uis3* and *LISP2*

**Figure 3:**
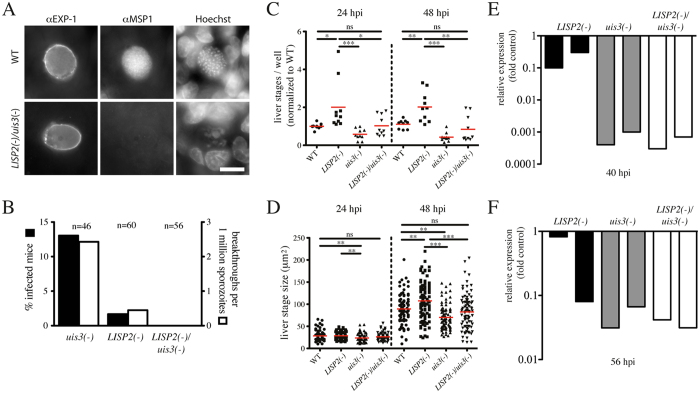
Characterization of a ANKA *LISP2*(–)*/uis3*(–) double knockout parasite line and comparison with single knockout lines. (**A**) Liver stages in HepG2 cells formed by WT and *LISP2*(–)*/uis3*(–) sporozoites 65 hours post infection as revealed by labelling with antibodies against EXP-1, and MSP1; Hoechst reveals host and parasite DNA. Scale bar: 10 μm. (**B**) Percentage of breakthrough infections (black) and infected mice per 1 million sporozoites injected (white) of the different parasite lines, see also Appendix Table SII. (**C**) Number of liver stages 24 and 48 hours post infection (hpi) of HepG2 cells with WT, *LISP2*(–), *uis3*(–) and *LISP2*(–)*/uis3*(–) sporozoites. All data normalized to the mean of WT duplicates in each individual experiment. Raw data are shown in Supplementary Fig. S4. (**D**) Sizes of liver stages 24 and 48 hours post infection (hpi) of HepG2 cells with WT, *LISP2*(–), *uis3*(–) and *LISP2*(–)*/uis3*(–) sporozoites. (**E,F**) Relative liver load of two mice 40 and 56 hours post infection (hpi) of *LISP2*(–), *uis3*(–) and *LISP2*(–)*/uis3*(–) sporozoite infected C57BL/6 mice. 18S rRNA abundance was normalized to the average value of 2 *Pb*ANKA infected mice for each time point. Note that at 56 hpi some WT parasites have already emerged from the liver, hence the lower relative levels.

We next generated a parasite line lacking the genes *uis3* and *LISP2* (Supplementary Fig. S3). These ANKA *LISP2*(–)/*uis3*(–) parasites showed no phenotypic or growth differences until the liver stage (data not shown). They readily infected HepG2 hepatocytes *in vitro* and developed into large liver stages but like the parasites lacking the individual genes never formed recognizable merozoites and showed little MSP1 staining (Fig. 3A). To assess the rate of breakthrough infection we infected 56 mice intravenously with a total of almost 5 million ANKA *LISP2*(–)/*uis3*(–) sporozoites. None of these mice developed blood stage parasitemia over the observation time of up to 150 days (Supplementary Table S2, Fig. 3B) suggesting a complete arrest of parasite development in the liver. These parasites also conferred complete protection from wild type challenge in a 3-shot regime (Table 1).

To investigate potential subtle differences in the protective capacity of the different strains we additionally tested all parasite lines in a single (one-shot) and a double (2-shot) immunization trial. For the one-shot trial we injected either 20.000 or 30.000 sporozoites of the respective parasite lines intravenously into mice and challenged these animals with 10.000 wild type sporozoites 14 days later. Curiously, this revealed that mice immunized with ANKA *LISP2*(–) parasites consistently showed a better protection than mice immunized with ANKA *uis3*(–) or the ANKA *LISP2*(–)*/uis3*(–) parasites (Tables 2 and 3). This suggests that ANKA *uis3*(–) parasites are not as protective as ANKA *LISP2*(–) parasites and that this *uis3*(–) phenotype is dominant in the parasites lacking both genes. Intriguingly, we found higher numbers of infected HepG2 cells and larger liver stages for ANKA *LISP2*(–) compared to WT parasites (Fig. 3C,D). In contrast, we found lower numbers of ANKA *uis3*(–) infected HepG2 cells at 24 and 48 hours post infection and smaller liver stage parasites. In the ANKA *LISP2*(–)*/uis3*(–) parasites both numbers of infected cells and liver stage size were comparable to WT. qRT-PCR analysis of infected livers after injection of 10.000 sporozoites showed a small decrease of 18S rRNA in ANKA *LISP2*(–) infected mice 40 and 56 hours post infection, while 18S rRNA in ANKA *uis3*(–) and *LISP2*(–)*/uis3*(–) infected livers was barely detectable (Fig. 3E,F). This might explain the decreased efficacy of ANKA *uis3*(–) and ANKA *LISP2*(–)*/uis3*(–) parasites compared to ANKA *LISP2*(–) parasites.

## Discussion

Our work revealed several interesting and relevant findings about ANKA *LISP2*(–) and *uis3*(–) parasites. Two previous papers have addressed the function of LISP2 by only partially disrupting the *LISP2* gene (Fig. 2A) resulting in a severe reduction of liver stage growth but not in a complete liver stage arrest^26^,^27^. Our work revealed an almost complete liver stage developmental arrest of the ANKA *LISP2*(–) parasites and showed that the N-terminus plays an important role in liver stage biology. The 403 amino acid long N-terminal region is conserved (>60% identity) within rodent malaria species (*P. berghei*, *P. yoelii* and *P. chabaudi*) and within the human parasite species *P. vivax* and *P. knowlesii* (Supplementary Fig. S1). However, it is rather diverse (<20% identity) between rodents and human species and absent in *P. falciparum*. This N-terminal region contains a PEXEL motif (aa 373–377 in *Pb*LISP2), which might play a role in secretion of the protein. Curiously our ANKA *LISP2*(–) parasites grew to larger size than the wild type *in vitro*, while they did not express large amounts of MSP1 (Figs 2D and 3D). This suggests that the size of the parasite and the developmental stage are not necessarily linked. The larger size might be due to the lack of merozoite formation and possibly an excess of membrane being deposited in the plasma membrane thus leading to a larger size, although this is currently just speculation.

Deletion of *uis3* in *P. berghei* strain NK65 led to an early arrest during liver stage development and attenuated parasites^9^. In contrast, the *P. berghei* strain ANKA *uis3*(–) parasite arrests later in what might be called a mid-stage (Fig. 3D) and causes breakthrough infections (Fig. 3B, Supplementary Table 2). Part of this difference might be due to a faster liver stage development of *P. berghei* ANKA parasites^29^,^30^. A clearer definition of the timing of arrest, also for the other parasite lines, should take into account the parasite size, the developmental stage of arrest as can be determined by electron microscopy and a gene expression profile.

Although vaccination with radiation-attenuated sporozoites (RAS) is still considered the gold standard for experimental malaria immunization (Seder *et al*.^3^), a first clinical trial demonstrated partial safety of *Pf*GAP-vaccination in humans^31^. Compared to RAS, GAPs arrest at more uniform time points during intrahepatic development^2^,^32^ either early^9^,^33^,^34^ or late^13^,^35^. Protective immunity appears to rely on CD8^+^ T cells and IFN-γ as key mediators of protection in all the different attenuation models^28^,^36^. However a GAP leading to an arrest in late liver stage development appears to elicit a larger number of CD8^+^ memory T cells with a broader antigenic repertoire as compared to early arresting GAP and/or RAS^15^. Whether this can be generalized to all GAPs arresting at the late liver stage however, remains to be determined. It is also not clear how the broader antigen repertoire and the increased size of the late GAP individually contribute to increased protection. The enhanced immune responses induced by these late arresting parasites correlate with enhanced protection suggesting that growth arrest during late liver stage development may confer superior protection than earlier arresting attenuated parasites^15^. As some single and double gene deletions can cause breakthrough infections^16^,^27^,^37^, it is important to find novel candidate genes that can be included in an attenuated parasite line lacking several genes as shown by various groups^21^,^22^,^24^,^36^. For example, early arresting NK65 *uis3*(–) parasites cause no breakthrough, while NK65 *uis4*(–) parasites show frequent breakthroughs. The mutant lacking both genes causes no breakthroughs and results in complete protection after immunizations, as do both individual *uis3*(–) and *uis4*(–) in a 3-shot regimen. However it is not clear if these multi-gene deletions affect protective efficacy.

Our data on 3 different parasite lines, which arrest during liver stage development showed a similar reduction in breakthrough infections but revealed interesting differences in their capacity to induce protective immune responses. Both ANKA *uis3*(–) and *LISP2*(–) parasites caused breakthrough infections during the immunization experiments. To quantify the rate of breakthrough we injected in total over 9 million sporozoites into over 160 mice (Fig. 3B, Supplementary Table S2). While our breakthrough rates are not problematic for experimental approaches similar rates in *P. falciparum* would clearly prevent use in human trials, let alone wider applications. To estimate if there is an additive safety effect of deleting both genes, we injected over 4.8 million of the *LISP2*(–)*/uis3*(–) sporozoites in 56 mice (Fig. 3B, Supplementary Table S2). In these mice we observed no breakthrough infections. This suggests that deletion of two genes leading to mid-stage arrest and possibly be involved in different biological functions can indeed add an additional level of safety as has been suggested previously for genes causing early arrest^31^. We would expect that in the best case this scaling would be the product of the infection rates of the individual KOs. Hypothetically this would result in an expected breakthrough rate of one per trillion sporozoites, which is far beyond the testable range. Ironically, this implies that the relationship of additive protection is most easily assessed with parasites showing very high breakthrough rates.

To test efficacy we turned to different vaccination schemes that generally lower protection from challenge. In a simple ‘one-shot’ immunization scheme, where mice were immunized just once and then challenged 14 days later with 10.000 wild type sporozoites, mice immunized with *LISP2*(–) parasites were better protected against wild type challenge than mice immunized with either *uis3*(–) or the *LISP2*(–)*/uis3*(–) sporozoites (Table 3). Similar data were obtained in ‘2-shot’ vaccination schemes (Table 2). These differences suggest a possible trade-off between achieving a fully save genetically attenuated parasite and a maximally protective one as the less protective gene deletion appears to dominate the efficacy of protection. This suggests, that the strains, while equally effective at establishing a hepatocyte infection, differ in their capacity to elicit protection during vaccination. A similar finding with just one gene deleted was shown for early arresting *uis3*(–) and *uis4*(–) *P. yoelii* parasites infecting BALB/c mice^28^. While *uis3*(–) but not *uis4*(–) *P. berghei* NK65 showed complete block in liver stage development^9^,^16^, deletion of either gene in *P. yoelii* led to a complete arrest^28^. Yet, immunization with *uis4*(–) *P. yoelii* parasites was more protective than with *uis3*(–) parasites^28^. However, there is no observable correlation in these experiments between time/safety of arrest, and efficacy of protection.

We suggest that many more genes that, upon deletion, arrest parasite development at defined points during infection should be identified. Their combination in multiple knockouts should then be tested to see which achieve totally abrogation of breakthrough events while keeping maximum protective efficacy. These should be tested in similar assays as shown here and also in assays that correlate the overall number of sporozoites used per immunization with protective capacity. Once more strains are available a careful dissection of the exact time of arrest in developmental terms as well as an immunological dissection of the different protective efficacies should be determined. Furthermore, alternative approaches to generate parasites that are attenuated at the liver stage such as the expression of foreign toxins^38^ or the induction of DNA double strand breaks^39^ should also be explored and tested towards their protective capacity prior to translation to *P. falciparum*.

## Materials and Methods

### Animals and parasites

C57BL/6 and NMRI mice were purchased from Charles River Laboratories. For all experiments six to eight weeks old female mice were used unless stated otherwise. *P. berghei* ANKA parasites were maintained by passaging *via* intra-peritoneal injections in NMRI mice and *Anopheles stephensi* mosquitoes. All animal experiments were performed according to FELASA standard guidelines and were approved by German authorities (Regierungspräsidium Karlsruhe).

### Generation of *LISP2*(–), *uis*3(–) and *LISP2*(–)/*uis3*(–) *P. berghei* ANKA mutants

To delete the genes encoding *LISP2* (Gene ID: PBANKA_100300) and *uis3* (Gene ID: PBANKA_140080) in *P. berghei* ANKA, the corresponding 5′UTR and 3′UTR regions were amplified from genomic DNA of mixed blood stages by polymerase chain reaction (PCR) using Phusion^®^ High-Fidelity DNA Polymerase (NEB, USA). For primers see Supplementary Table S1. The amplified fragments of *LISP2* were ligated into Pb262 vector that contains the human dihydrofolate reductase and the yeast bifunctional enzyme cytosine deaminase as well as uracil phosphoribosyl transferase (hdhfr-yFCU), as a positive-negative selection cassette^17^. The 5′UTR and 3′UTR fragments of *uis3* were ligated into the b3D-vector containing the *h*DHFR resistance cassette. The linearized vectors were transfected into *P. berghei* ANKA merozoites using the Nucleofector™ kit in the Amaxa (Lonza) electroporator as previously described^40^ followed by intravenous injection into NMRI mice. Mutant parasites were selected by adding pyrimethamine (0.07 mg/ml, Sigma-Aldrich) to the drinking water (*PbANKA LISP2*(–)) or by subcutaneous injection of WR99210 (16 mg/ kg body weight, Sigma-Aldrich) (*PbANKA uis3*(–)) and clones were derived by limiting dilution. The integration of the vector into the genome was confirmed by PCR using test primer pairs as shown in the figures and Supplementary Table S1. Two different transfections were performed to obtain independent clones for *LISP2*(–) and *uis3*(–) ANKA parasites. Negative selection of *Pb* ANKA *LISP2*(–) parasites was performed by adding 5-Fluorocytosine (1 mg/ml, Sigma-Aldrich) in the drinking water. The *LISP2*(–)*/uis3*(–) ANKA parasite line was generated by transfecting the linearized *uis3*(–) knock-out vector (b3D containing *Tg*DHFR resistance cassette) into a negatively selected *LISP2*(–) parasite line. Similarly the N-terminus of *LISP2* was integrated into a negatively selected *LISP2*(–) parasite line using Pb238 vector containing the corresponding 5′UTR and 3′UTR regions flanking the *h*DHFR resistance cassette.

### Mosquito stage development and sporozoite infectivity

Sporozoite numbers were determined in the midguts and salivary glands by mosquito dissection 12 and 17/18 days post feeding, respectively. Gliding motility of sporozoites was analyzed in glass bottom 96 well plates (Greiner) using freshly dissected salivary gland sporozoites as described before^41^.

Confluent HepG2 cells were infected with freshly isolated salivary gland sporozoites, cultivated in DMEM supplemented with 10% fetal calf serum, 1 mM glutamine and 1% Anti-anti (Gibco) at 37 °C and 5% CO_2_ and fixed with 4% paraformaldehyde followed by ice cold methanol. Primary antibodies used for staining: monoclonal mouse anti-*Pb*HSP70^42^, rat anti-*Pb*MSP1^43^, mouse anti-*Pb*EXP1; secondary antibodies: Alexa-Fluor® 488 goat anti-mouse IgG antibody, Alexa-Fluor® 546 goat anti-rat IgG antibody; DNA was revealed by Hoechst 33342 (all Life Technologies). Size measurements were carried out by acquisition of images with a Zeiss Axiovert 200 M microscope followed by analysis using Volocity (PerkinElmer).

### *In vivo* infectivity of mutant sporozoites and immunizations

10 infected mosquitoes (on day 17 post infection) were allowed to bite anaesthetized mice or isolated salivary gland sporozoites in 100 μL PBS were injected intravenously per mouse. Parasitemia was monitored by daily Giemsa stained blood smears starting at day 3 after infection.

For immunization C57BL/6 mice were intravenously injected with sporozoites according to the different immunization protocols described in Tables 1–3. Immunized mice were monitored for absence of blood stage parasites by daily Giemsa stained blood smears. Challenge of all immunized and naïve control mice with wild type *Pb* ANKA sporozoites was performed as depicted in Tables 1–3. Blood stage parasitemia was monitored from day 3 onward up to 21 days after the challenge.

### Real-time PCR analysis

C57BL/6 mice were infected by intravenous injection of 20.000 sporozoites of the respective parasite strain. 40 h or 56 h post-infection mice were sacrificed, livers were homogenized in 4 ml QIAzol (QIAGEN) and 0.5 ml aliquots were frozen at –80 °C immediately. RNA extraction followed by DNA digest using the TURBO DNA-free Kit (Ambion) was performed according to the manufacturer’s instructions. cDNA was synthesized with the First Strand cDNA Synthesis Kit (ThermoFisher) and checked afterwards for gDNA contaminations via RT-PCR. Real-time PCR was performed in real time using triplicates on a CFX96 Touch Real-Time PCR Detection System (BIO-RAD) using Power SYBR Green PCR Master Mix (Applied Biosystems) and gene-specific primers (P18-21, see Supplementary Table S1). Relative copy numbers of the different parasite lines were calculated by applying the ∆∆C_t_ methodology.

### Statistical analysis

1-way ANOVA with Bonferroni post test was performed with Prism software (version 5.0b, GraphPad). A p-value of <0.05 was considered significant.

## Additional Information

**How to cite this article**: Kumar, H. *et al*. Protective efficacy and safety of liver stage attenuated malaria parasites. *Sci. Rep*. **6**, 26824; doi: 10.1038/srep26824 (2016).

## Supplementary Material

Supplementary Information

## Figures and Tables

**Table 1:** Protection of C57BL/6 mice immunized in a 3-shot regimen with the indicated strains against challenge with wildtype *P. berghei* ANKA sporozoites or blood stages).

Exp.	Attenuated parasite line (*P. berghei* ANKA)	1^st^ shot	2^nd^ and 3^rd^ shots* (day of shots)	Challenge dose# (day of challenge)		Protected/challenged animals (pre-patency)+
				Challenge 1	Challenge 2	
1	*LISP2*(–)	10.000	10.000 (d14, d28)	1.000 (d42)	10.000 (d116)	C1: 3/4 (d 6), C2: 1/1
					5.000 bs (d116)	C2: 0/2 (d 4.5)
				1.000		0/4 (d 3)
2	*LISP2* (–)	50.000	20.000 (d14, d28)	10.000 (d42)	10.000 (d102)	C1 : 4/4, C2 : 2/2
					10.000 bs(d102)	C2 : 0/2
	*uis3*(–)	50.000	20.000 (d14, d28)	10.000 (d42)	10.000 (d102)	C1: 6/6**, C2: 6/6
	*LISP2*(–)*/uis3*(–)	50.000	20.000 (d14, d28)	10.000 (d42)	10.000 (d102)	C1: 8/8, C2: 8/8
				10.000		0/8 (d 3.1)

Groups of C57BL/6 age-matched mice were used for different immunizations. Blood stages were included to probe stage specific protection.

^*^Data are presented as numbers of sporozoites for first, second and third shot; days of shots are indicated in parentheses.

^#^mice are challenged with *Pb*ANKA wild type sporozoites or blood stages (bs); days of challenge after prime are indicated in parentheses.

^+^C1: challenge 1, C2: challenge 2; day of blood stage emergence (pre-patency) is indicated in parentheses.

^**^Two mice became positive after priming; thus challenge was only performed with 6 mice.

**Table 2:** Protection of C57BL/6 mice immunized in a 2-shot regimen with the indicated *P. berghei* ANKA knockout lines against challenge with wild type *P. berghei* ANKA sporozoites or blood stages.

Exp.	Attenuated parasite line (*P. berghei* ANKA)	1^st^ shot	2^nd^ shot* (day14)	Challenge dose# (day of challenge)	Protected/challenged animals (pre-patency)
3	*LISP2*(–)	10.000	10.000	1.000 (d42)	1/4 (d 5.75)
				1.000 (d42)	0/4 (d 3)
4	*LISP2*(–)	20.000	20.000	10.000 (d28)	8/8
	*uis3* (–)	20.000	20.000	10.000 (d28)	3/7** (8.75 d)
	*LISP2*(–)*/uis3*(–)	20.000	20.000	10.000 (d28)	3/8 (8.6 d)
				10.000 (d28)	0/4 (3.75 d)

^*^Numbers of sporozoites for first boost at day 14; d0 corresponds to date of first shot.

^#^Mice were challenged with *Pb*ANKA wild type sporozoites; day of challenge after prime in parentheses.

^**^1 breakthrough infection after priming with 20.000 *Pb*ANKA *uis3*(–) sporozoites.

**Table 3:** Protection of C57BL/6 mice immunized in a 1-shot regime with the indicated *P. berghei* ANKA knockout lines against challenge with wild-type *P. berghei* ANKA sporozoites.

Exp.	Attenuated parasite line (*P. berghei* ANKA)	1^st^ shot	Challenge dose (day 14)	Protected/challenged animals (pre-patency)
5	*LISP2*(–)	30.000	10.000	7/15* (d6.25)
	*uis3* (–)	30.000	10.000	4/16 (d5)
	*LISP2*(–)*/uis3*(–)	30.000	10.000	0/16 (d5.1)
			10.000	0/16 (d3.2)
6	*LISP2*(–)	20.000	10.000	4/6 (d7)
	*uis3* (–)	20.000	10.000	0/4* (d6)
	*LISP2*(–)*/uis3*(–)	20.000	10.000	1/6 (d5.4)
			10.000	0/4 (d4.25)

^*^1 breakthrough infection after injection of 30.000 *LISP2*(–) sporozoites and 2 breakthrough infections after injection of 20.000 *uis3*(–) sporozoites.
