# Supplementary material for: Protective efficacy and safety of liver stage attenuated malaria parasites
Source: Sci Rep. 2016 May 31;6:26824. doi: 10.1038/srep26824 (PMC4886212; doi:10.1038/srep26824)
Supplement: Supplementary Information [file srep26824-s1.pdf]

Supplementary material for

Protective efficacy and safety of liver stage attenuated malaria parasites

Hirdesh Kumar, Julia Magdalena Sattler, Mirko Singer, Kirsten Heiss,
Miriam Reinig, Christiane Hammerschmidt-Kamper, Volker Heussler,
Ann-Kristin Mueller and Friedrich Frischknecht

Contents:

-Supplementary Fig. S1
-Supplementary Fig. S2
-Supplementary Fig. S3
-Supplementary Fig. S4
-Supplementary Table S1
-Supplementary Table S2

UIS3

full protein (upper half) / ETRAMP domain (lower half)

	Pf	Pv	Pk	Pb	Py	Pc
Pf		39	38	33	32	30
Pv	41		85	40	41	43
Pk	36	85		41	42	43
Pb	24	26	26		86	73
Py	23	27	28	84		70
Pc	24	28	28	70	66	

full protein (upper half) / UIS3 domain (lower half)

	Pf	Pv	Pk	Pb	Py	Pc
Pf		39	38	33	32	30
Pv	43		85	40	41	43
Pk	43	87		41	42	43
Pb	39	54	55		86	73
Py	39	55	55	87		70
Pc	36	61	59	79	78	

LISP2

full protein (upper half) / 6-Cystein domain (lower half)

	Pf	Pv	Pk	Pb	Py	Pc
Pf		21	21	29	26	25
Pv	48		51	25	24	24
Pk	46	75		25	25	23
Pb	49	47	47		80	67
Py	49	48	48	90		65
Pc	50	50	49	87	89	

full protein (upper half) / N-terminal 403 aa (lower half)

	Pf	Pv	Pk	Pb	Py	Pc
Pf		21	21	29	26	25
Pv			51	25	24	24
Pk		63		25	25	23
Pb		16	17		80	67
Py		16	18	87		65
Pc		17	18	76	76	

Figure S1: Percentage identity matrices of UIS3 and LISP2 in different *Plasmodium* species.

Pf: *P. falciparum* 3D7; Pv: *P. vivax* Sal1; Pk: *P. knowlesii*; Pb: *P. berghei* ANKA; Py: *P. yoelii* 17X; Pc: *P. chabaudi chabaudi*. Dark cells represent sequences with >40% amino acid identity. Upper half matrix: full length proteins; lower half matrix: ETRAMP and UIS3 domains as well as 6-cystein domain and 403 amino acid N-terminal region. An N-terminal region in *P. falciparum* could not be identified.

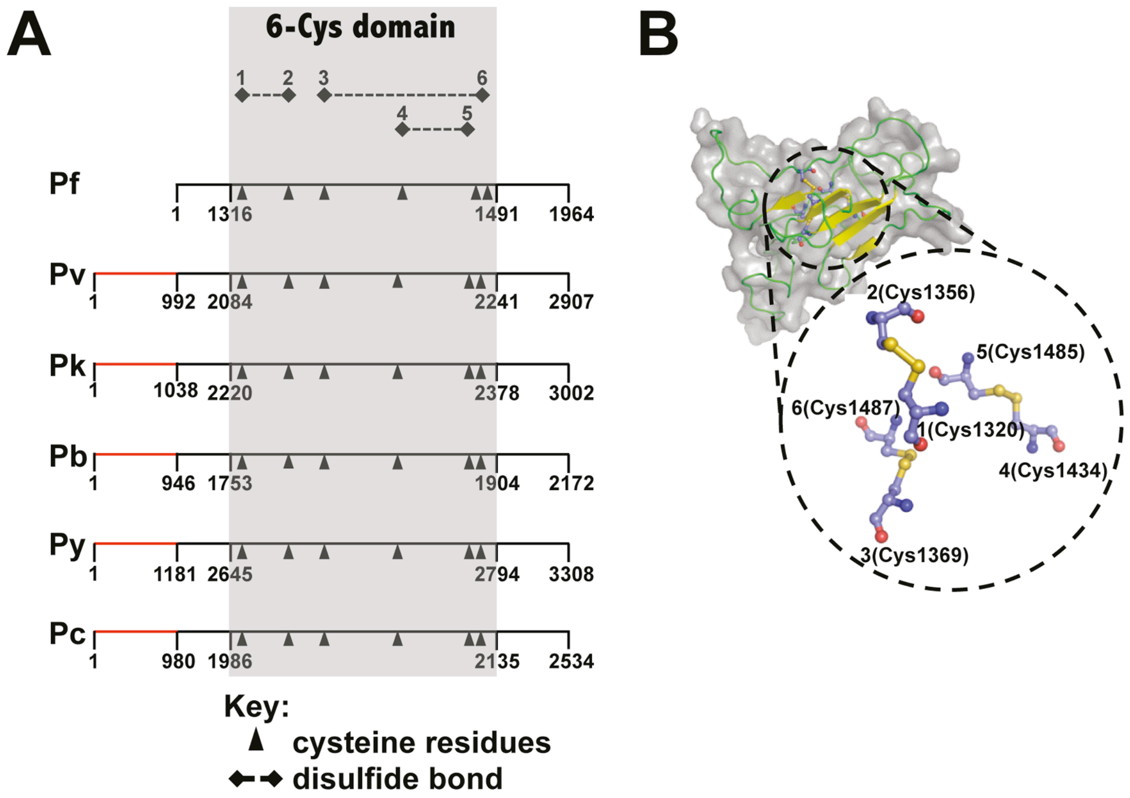

Figure S2: Predicted structure of the 6-cystein domain of LISP2.

(A) Representative layout of LISP2 amino acid sequences in different *Plasmodium* species (Pf: *P. falciparum*, Pv: *P. vivax*, Pk: *P. knowlesii*, Pb: *P. berghei*, Py: *P. yoelii*, Pc: *P. chabaudi*). The 6-Cys domains predicted using SMART server, is highlighted in grey. Numbers below the gene models indicate amino acid positions.

(B) Homology model of the 6-Cys domain in *Pf*LISP2 (corresponding to the grey region in B) predicted by BhageerathH server. The characteristic β-sandwich is shown in a ribbon diagram, overlapped with the surface view (grey). The predicted loop region is hidden for clarity. The enlarged view depicts 6-conserved cysteine residues (arrow head in B), forming 3 disulfide bonds.

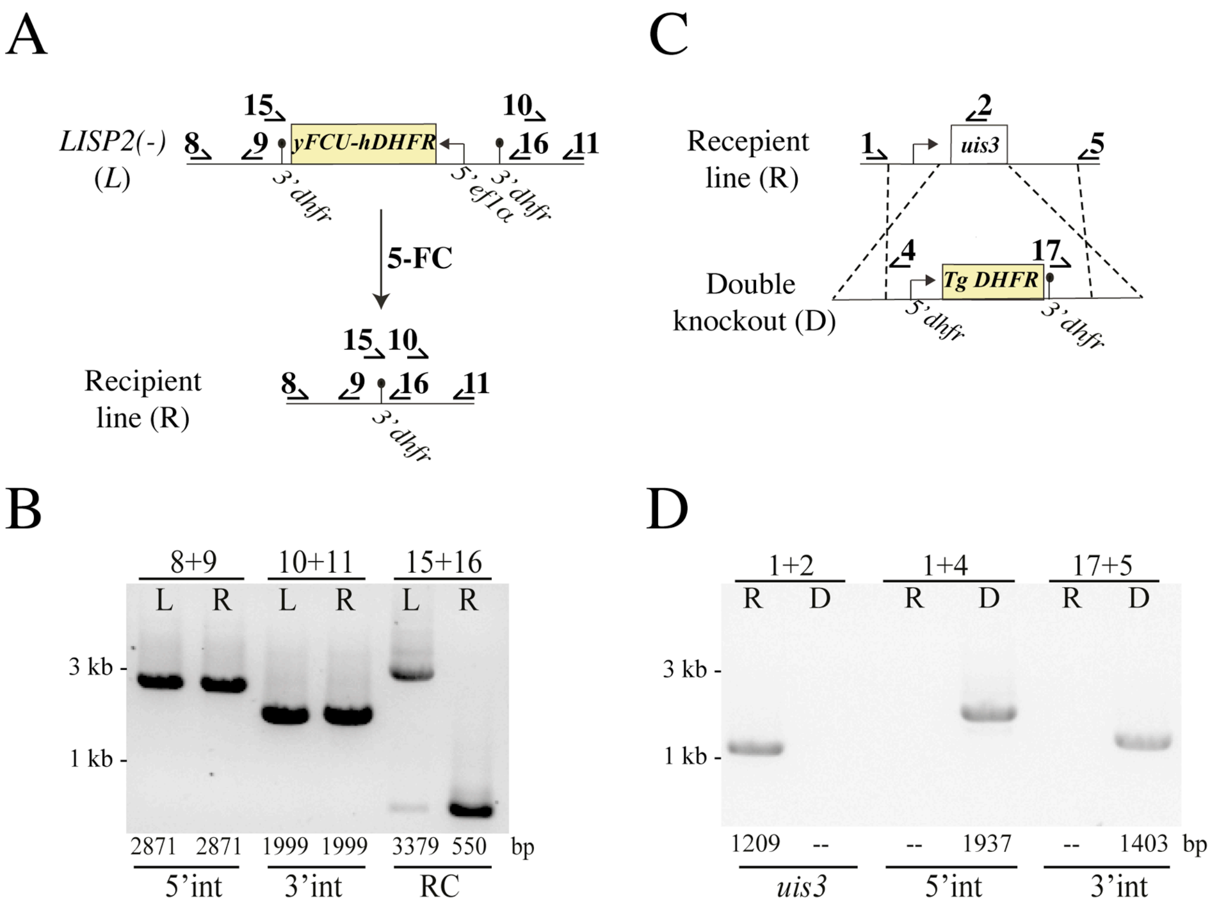

Figure S3: Negative selection of *LISP2(-)* parasites to remove resistance marker and generation of the *LISP2(-)/uis3(-)* double knockout parasite line.

(A) Schematic of the removal of the selection cassette by negative selection using 5-Fluorocytosine (5-FC). Primers used for diagnostic PCR reactions in B are indicated.

(B) Diagnostic PCR reactions and expected amplicon sizes.

(C) Schematic of the *uis3* deletion strategy using the 5' and 3'UTRs of *uis3* to integrate a resistance cassette by double crossover into the *LISP2(-)* parasite genome. The locations of primers used for PCR in D are indicated.

(D) Diagnostic PCR reactions and expected amplicon sizes.

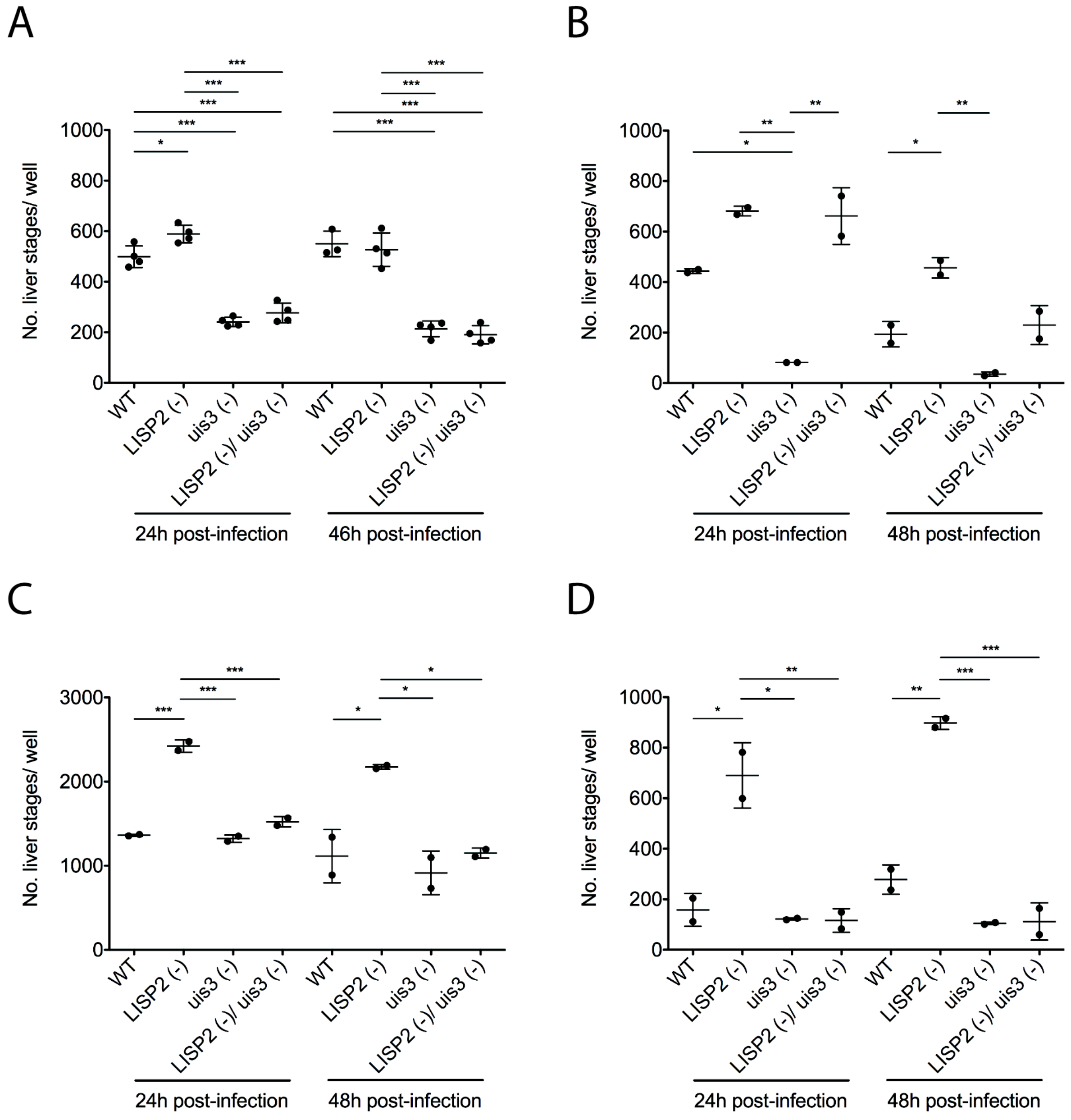

Figure S4: Characterization of a ANKA *LISP2(-)/uis3(-)* double knockout parasite line and comparison with single knockout lines in four individual liver stage assays.

(A) Number of liver stages per well 24 and 46 hours post infection (hpi) of HepG2 cells with WT, *LISP2(-)*, *uis3(-)* and *LISP2(-)/uis3(-)* sporozoites. Four individual wells were counted. Mean numbers (± SD) are shown.

(B-D) Number of liver stages per well 24 and 48 hours post infection (hpi) of HepG2 cells with WT, *LISP2(-)*, *uis3(-)* and *LISP2(-)/uis3(-)* sporozoites. Two individual wells per assay were counted. Mean numbers (± SD) are shown.

Supplementary Table 1: Primers used in this study.

Used for	No.		Restriction Enzymes	Sequence
*Pb*A *uis3* (-)				
5' UTR		uis3-5'f	*EcoRI*	CG**GAATTC**GCTAGCATTAGCATAACATCTCATTGGC
		uis3-5'r	*KpnI*	GG**GGTACC**GCATGCAACCACAACATAAACAAAATTAATG
3' UTR		uis3-3'f-	*PstI*	AA**CTGCAG**GCCTGTACACTATATTGTATATTAGC
		uis3-3'r	*HindIII*	CCC**AAGCTT**CCATCTACGCCTTTTATGCTT
Test-WT	1	Test-5'f	--	TGCTATAAAGCGTATTATGAATTAATATAAAAGG
	2	uis3- r	--	GTCCTGATGATAACAAAGCAATTGCAAC
Test-5'Int	1	Test-uis3-5'f	--	TGCTATAAAGCGTATTATGAATTAATATAAAAGG
	3	Test-uis3-5'r	--	GGCCCCGTTTTTCTTACTTATATATTTATACCAATTG
Test-3'Int	4	Test-uis3-3'f	--	GTCCATACAACTATATCCGAAC
	5	Test-uis3-3'r	--	GTATTTATGCCAAAAAAGCTAAATTGC
*Pb*A *lisp2 (-)*				
5'UTR		lisp2-5'f	*Sal1*	ATC**GTCGAC**TGGTATCCATTATAGCAGCAAACAC
		lisp2-5'r	*EcoR1*	GTCAC**GAATTC**AATTTGTTTGCACGCTTTATCG
3'UTR		lisp2-3'f	*HindIII*	ACCG**AAGCTT**TGTTTGGACATAATGAATACGATGG
		lisp2-3'r	*Xho1*	ACCG**CTCGAG**CCACGGATGCACTAATAGAAGC
Test-gene	6	lisp2-gene-f	NheI	AT**GCTAGC**ATGAAAGATCATATAAAAAATGTATGCTTTCG
	7	lisp2-gene-r	BamHI	AC**GGATCC**CGTGTTTCCTTTCTTTTTCTCG
Test-5'Int	8	Test-lisp2-5'f	--	TTGTGCACCCTCGTATACTG
	9	Test-lisp2-5'r	--	ACGCAACCTTATATTCCTCAATTAC
Test-3'Int	10	Test-lisp2-3'f	--	CATACTAGCCATTTTATGTG
	11	Test-lisp2-3'r	--	GAAGCAACTATGAAAAAACTAATGGG
RC	15	Test hdfr-r	--	GCCGATATCCAAGAAAGCTGGGTGGTACCC
	16	Test hdfr-f	--	CCCAAGCTTCAAAAAAGCAGGCTTGCCGC
N-LISP2				
Gene-insert	6	N-lisp2-f	*Nhe1*	AT**GCTAGC**ATGAAAGATCATATAAAAAATGTATGCTTTCG
	7	N-lisp2-r	*BamH1*	AC**GGATCC**CGTGTTTCCTTTCTTTTTCTCG
Promoter-insert		lisp2-Prom-f	*Xho1,Not1, SacII*	AAA**CTCGAGGCGGCCGC**AAA**CCGCGG**GAGTTGCATTATC GTCAAAAGTG
3'insert		lisp2-3-f	*HindIII*	AAA**AAGCTT**AGAGCTACCAAAATTTAAAGAAAATCC
		lisp2-3'r	*Xho1*	ACCG**CTCGAG**CCACGGATGCACTAATAGAAGC
Test-gene	6	N-lisp2-f	*Nhe1*	AT**GCTAGC**ATGAAAGATCATATAAAAAATGTATGCTTTCG
	7	N-lisp2-r	*BamH1*	AC**GGATCC**CGTGTTTCCTTTCTTTTTCTCG
Test-5'Int	8	Test-Nlisp2-5'f	--	TTGTGCACCCTCGTATACTG
	12	Test-N-lisp2-5'r	--	CGCAATTTGTTGTACATAAAATAGGC
Test-3'Int	6	Test-N-lisp2-3'f	*Nhe1*	AT**GCTAGC**ATGAAAGATCATATAAAAAATGTATGCTTTCG
	11	Test-lisp2-3'r	--	GAAGCAACTATGAAAAAACTAATGGG
RC	13	Test hdfr-r	--	GCC**GATATC**CAAGAAAGCTGGGTGGTACCC
	14	Test hdfr-f	--	CCC**AAGCTT**CAAAAAAGCAGGCTTGCCGC
*Pb*A *LISP2(-)/uis3(-)*				
Test-gene	1		--	TGCTATAAAGCGTATTATGAATTAATATAAAAGG
	2		--	GTATTTATGCCAAAAAAGCTAAATTGC
Test-5'Int	1		--	TGCTATAAAGCGTATTATGAATTAATATAAAAGG
	4		--	GTCCATACAACTATATCCGAAC
Test-3'Int	17		--	AAAGCTAGCTTTGATCCCGTTTTTC
	5		--	GTATTTATGCCAAAAAAGCTAAATTGC

Supplementary Table 2: Total number of i.v. injected sporozoites and mice.

**Attenuated parasite line (*P. berghei* ANKA)**	**Mice***	**Injected sporozoites**	**Break-through infections**	**Total number of injected sporozoites**
LISP2(-)	15	10.000	-	2.230.000
	14	20.000	-	
	17	30.000	1	
	12	50.000	-	
	1	220.000	-	
	1	470.000	-	
uis3(-)	14	20.000	3	2.470.000
	16	30.000	-	
	12	50.000	3	
	3	220.000	-	
	1	450.000	-	
LISP2(-) /*uis3*(-)	14	20.000	-	4.870.000
	16	30.000	-	
	9	50.000	-	
	1	100.000	-	
	7	200.000	-	
	3	220.000	-	
	6	250.000	-	
N-LISP2	4	10.000	4	160.000
	4	30.000	4	

*all mice injected with sporozoites were naive
